# Gut Bacteria Improve Depressive Symptoms by Degrading Cortisol into Androgen

**DOI:** 10.1002/advs.202508468

**Published:** 2026-01-08

**Authors:** Xiong Wang, Qing Wu, Hao‐Long Zeng, Yin Shen, Hong‐Han Zhang, Jun Gong, Qi Zhang, Jia‐Zhao Xie, Da‐Wei Ye, Zi‐Yong Sun, Zhong‐Chun Liu, Li‐Ming Cheng, Di Li

**Affiliations:** ^1^ Department of Laboratory Medicine Tongji Hospital Tongji Medical College and State Key Laboratory for Diagnosis and Treatment of Severe Zoonotic Infectious Diseases Huazhong University of Science and Technology Wuhan Hubei 430030 China; ^2^ Department of Laboratory Medicine Tongji Hospital Tongji Medical College Huazhong University of Science and Technology Wuhan Hubei 430030 China; ^3^ Department of Laboratory Medicine institute of translational medicine, Renmin Hospital of Wuhan University Wuhan Hubei 430060 China; ^4^ Department of Psychiatry Renmin Hospital of Wuhan University, Taikang Center for Life and Medical Sciences, State Key Laboratory of Metabolism and Regulation in Complex Organisms, Wuhan University Wuhan Hubei 430060 China; ^5^ Department of Biliary‐Pancreatic Surgery Tongji Hospital Tongji Medical College Huazhong University of Science and Technology Wuhan Hubei 430030 China; ^6^ Department of Plastic and Cosmetic Surgery Tongji Hospital Tongji Medical College Huazhong University of Science and Technology Wuhan Hubei 430030 China; ^7^ Precision Medical Center Wuhan Children's Hospital (Wuhan Maternal and Child Healthcare Hospital) Tongji Medical College Huazhong University of Science and Technology Wuhan 430015 China; ^8^ Cancer Center Tongji Hospital Tongji Medical College Huazhong University of Science and Technology Wuhan 430030 China

**Keywords:** cortisol, depression, gut microbiota

## Abstract

Gut microbiota may play a role in regulating the stress hormone cortisol. However, the mechanisms underlying the regulation of cortisol by gut microbiota are poorly understood. Here, it is found that gut microbiota degraded cortisol, and this ability is heterogeneous among individuals. Mice gavaged with gut microbiota having a low ability to degrade cortisol may be prone to developing depressive‐like behavior. The cortisol‐degrading strain Pseudomonas aeruginosa Tongji is isolated from faecal microbiota. The des‐like enzyme expressed by P. aeruginosa Tongji converts cortisol into androgen. Bacterial des‐like enzyme protects mice against depressive‐like behavior via degrading cortisol and producing androgen. The probiotic Bacillus subtilis expressing the des‐like enzyme is constructed, which protects the host against cortisol elevation. These findings suggest that bacterial des‐like enzyme may regulate cortisol and improve depression by converting cortisol into androgen.

## Graphical abstract

Chronic stress is an important risk factor for stress‐related disorders such as depression. Stress hormone cortisol is essential for the pathogenesis of stress‐related disorders such as depression. Some gut microbiota degraded cortisol, and improve depressive symptoms. Probiotic expressing the cortisol‐degrading enzyme protected host against cortisol elevation is an effective intervention measure for depression.

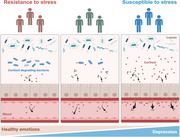

## 1 Introduction

Chronic stress, such as poverty, chronic somatic illness or enduring social conflicts, is an important risk factor for stress‐related disorders such as depression.^[^ ^1^ ^]^ Approximately 92% of patients with depression encounter stressful events before disease onset.^[^ ^2^ ^]^ The hypothalamic–pituitary–adrenal (HPA) axis is activated in response to stress, primarily via the steroid hormone cortisol.^[^ ^3^ ^]^ Chronic cortisol exposure can lead to depression,^[^ ^4^ ^]^ and ≈20–80% of individuals with depression present with elevated cortisol levels.^[^ ^5^ ^]^ Among those who encounter traumas or tragedies, 10–30% manifest symptoms of depression.^[^ ^6^ ^]^

Understanding the mechanisms underlying the regulation of cortisol is crucial for the development of preventive and therapeutic strategies.

Previous studies have shown that a reduction in the gut microbiota of rodents increases the levels of the stress hormone. For example, germ‐free mice experience increased stress hormone levels after long‐term exposure to restraint stress.^[^ ^7^ ^]^ Moreover, germ‐free rodents exhibit increased levels of corticosterone under various stressful conditions, such as social interaction^[^ ^8^ ^]^, maternal separation,^[^ ^9^ ^]^ bacterial endotoxin injection,^[^ ^10^ ^]^ environmental change,^[^ ^11^ ^]^ and open field testing.^[^ ^12^ ^]^ Similar conclusions were drawn from mouse experiments using antibiotics that eradicate gut microbiota. A recent preliminary study in rodents suggested gut microbiota may play a role in regulating the stress hormone. ^[^ ^8^ 8 ^]^ However, how gut microbiota regulate cortisol remains unclear.

## 2 Results

### 2.1 The Ability of Gut Microbiota to Regulate Cortisol Varies among Individuals

**Figure 1:**
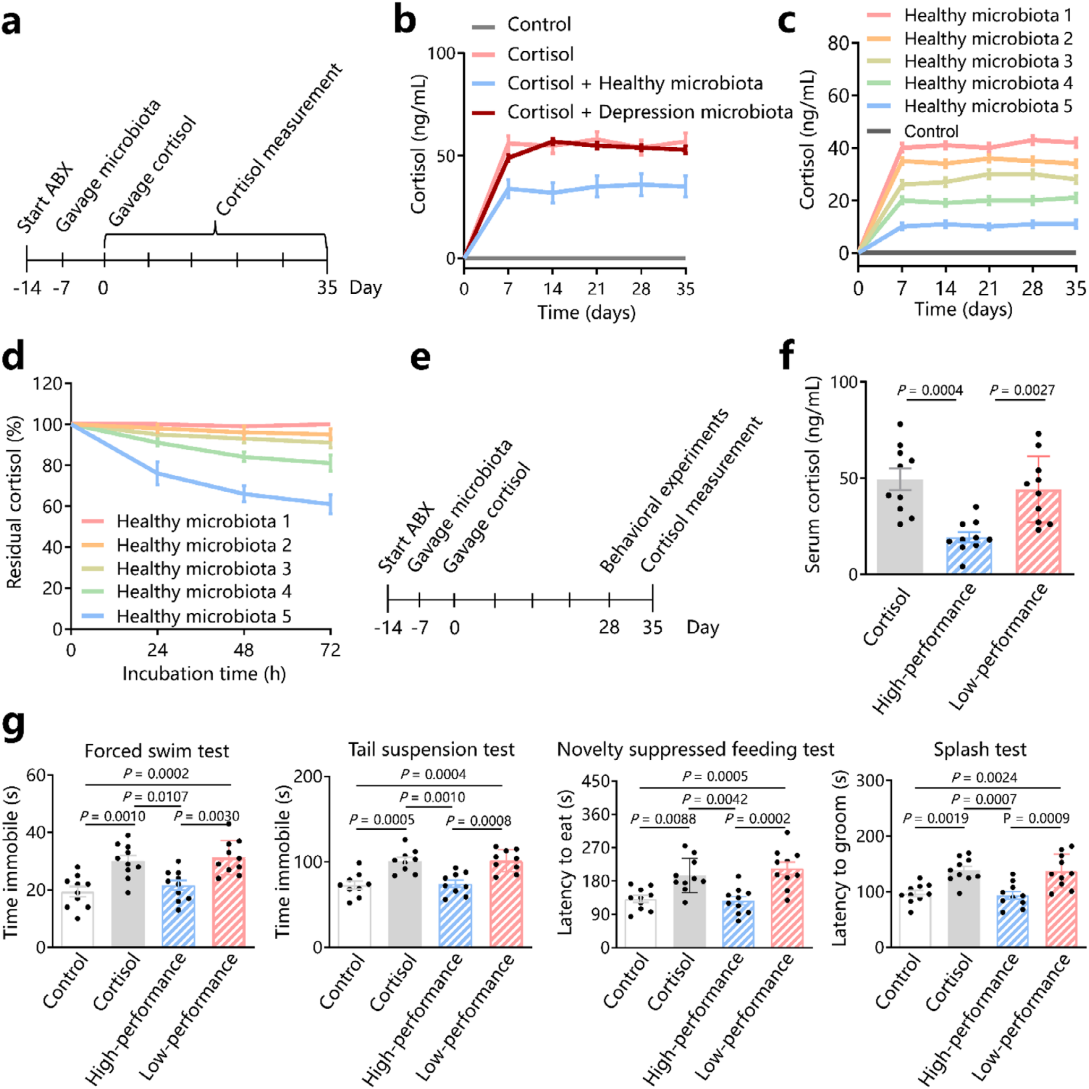
Gut microbiota protects against increases in cortisol levels and depressive‐like behaviors. a) C57BL/6 mice were pre‐treated with antibiotics (ABX) for 7 days, followed by microbiota gavage (once per day for three successive days every week for 4 weeks) and subjected to cortisol for 4 weeks. Serum cortisol levels were measured every week. b) Serum cortisol levels in control mice (control group), cortisol‐treated mice (cortisol group), mice gavaged with microbiota from healthy individuals (healthy microbiota group), and mice gavaged with patients with depression (depression microbiota group). c) Serum cortisol levels in control mice and mice gavaged with microbiota from five healthy participants, respectively. d) Cortisol levels incubated in vitro with microbiota derived from five healthy participants, respectively. e) C57BL/6 mice were pre‐treated with ABX for 7 days, followed by microbiota gavage (once per day for three successive days every week for 4 weeks) and cortisol treatment for 28 days. f) Serum cortisol levels in mice treated with cortisol (cortisol group), mice gavaged with microbiota that have high performance in degrading cortisol (high‐performance group), and mice gavaged with microbiota that cannot degrade cortisol (low‐performance group). g) Behavioral experiments. Data are representative of at least three independent experiments, *n* = 10 mice per group. Data are mean ± S.D. One‐way ANOVA followed by post hoc Tukey multiple comparison test.

To explore the effects of the gut microbiota on cortisol, we investigated the effects of microbiota transplantation on the levels of cortisol in mice exposed to cortisol (**Figure** **1**a). In cortisol‐treated mice, cortisol was detected in the gut, liver and brain to enhance the understanding of systemic cortisol dynamics. We transplanted faecal microbiota from healthy individuals (*n* = 5) or patients with depression (*n* = 5) into randomized mice via gavage. The microbiota were mixed from the 5 people in this experiment. Based on liquid chromatography‐tandem mass spectrometry (LC‐MS/MS) analyses, the cortisol levels of mice that received faecal microbiota from patients with depression did not significantly differ after cortisol treatment from those in the cortisol group (Figure 1b). By contrast, mice that received faecal microbiota from healthy volunteers had lower cortisol levels after cortisol treatment than mice in cortisol group (Figure 1b). These results are similar to the findings of previous research,^[^ ^13^ ^]^ indicating that the gut microbiota from healthy individuals protects against the elevation of cortisol levels.

We gavaged the gut microbiota of each healthy individual into a group of mice to investigate whether the protective effects of faecal microbiota against cortisol elevation differ among different healthy individuals. After cortisol treatment, LC‐MS/MS showed that the faecal microbiota of the five healthy individuals induced varying degrees of protection against the elevation of cortisol levels. The cortisol levels did not significantly differ between mice who received human microbiota that conveyed the worst protection and those in the cortisol group (Figure 1c). The cortisol levels of mice that received human microbiota with the most effective protection are close to those in the control group (Figure 1c). These results suggest that the ability of gut microbiota to regulate cortisol levels varies among individuals.

### 2.2 Gut Microbiota‐Regulated Cortisol Depends on the Ability of Cortisol Degradation

We speculated that the protective effect of faecal microbiota on elevated cortisol levels is related to the ability of the bacteria to degrade cortisol. To investigate whether the gut microbiota of healthy individuals can degrade cortisol, we treated cultures of the faecal microbiota of the healthy individuals with high concentrations of cortisol (1 g L^−1^). Interestingly, the faecal microbiota of the five healthy individuals exhibited varying degrees of cortisol degradation ability in vitro. The gut microbiota of healthy volunteers with the strongest degradation ability degraded cortisol efficiently, whereas the gut microbiota with the weakest degradation ability did not show significant cortisol degradation (Figure 1d). Moreover, cortisol showed no significant degradation after incubation with the faecal microbiota from patients with depression. These results indicate that the faecal microbiota of healthy volunteers can degrade cortisol, and this degradation ability varies among individuals.

To investigate whether the gut microbiota‐regulated cortisol was related to the cortisol degradation ability, we gavaged mice with the faecal microbiota of healthy volunteers with the strongest (high‐performance group) and no (low‐performance group) ability to degrade cortisol (Figure 1e). The serum cortisol level of mice in the low‐performance group did not significantly differ from that in the cortisol group (44.2 ± 17.20 vs 49.3 ± 17.7, *p* > 0.05) (Figure 1f). The high‐performance group had lower levels of circulating cortisol than the cortisol group (19.3 ± 8.43 vs 49.3 ± 17.7, *p* = 0.0004) (Figure 1f). These results indicate that the ability of the gut microbiota of healthy volunteers to protect against elevated levels of circulating cortisol in the host is related to their ability to degrade cortisol. Mice with gut microbiota lacking the ability to degrade cortisol are more prone to develop elevated cortisol levels.

Next, we conducted behavioral experiments on mice treated with gut microbiota with different cortisol degradation abilities to investigate the effects on behavior. Similar to previous research results,^[^ ^14^, ^15^ ^]^ mice that received cortisol exhibited higher immobile and latency time in the splash test, novelty‐suppressed feeding test (NSFT), forced swimming test (FST), and tail suspension test (TST) (*p* < 0.05), indicating depressive‐like behavior (Figure 1g). Compared with mice in the control group, mice in the high‐performance group did not exhibit depressive‐like behavior, whereas those in the low‐performance group did (Figure 1g). These results indicate that the ability of the gut microbiota to protect against cortisol‐induced depressive‐like behavior is related to the cortisol‐degrading effect of the microbiota. Mice with gut microbiota lacking the ability to degrade cortisol are susceptible to depressive‐like behaviors.

### 2.3 The Cortisol‐Degrading Bacterium *Pseudomonas aeruginosa* Tongji Protects against Cortisol Elevation and Depressive‐Like Behavior in Mice

**Figure 2:**
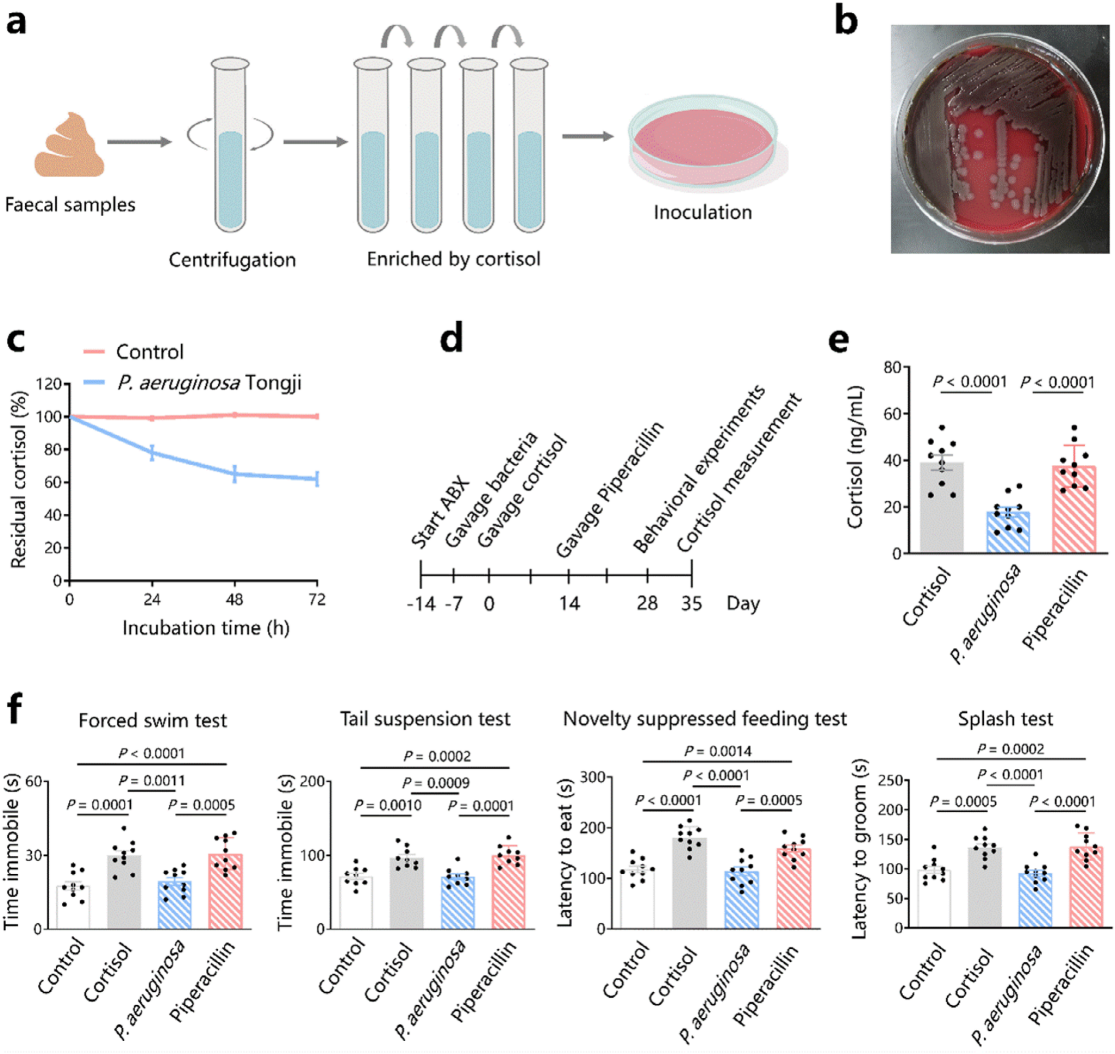
Cortisol‐degrading *P. aeruginosa* Tongji protects against increases in cortisol levels and depressive‐like behavior. a) Schematic presentation of cultivation, enrichment, and isolation of cortisol‐degrading bacteria from faecal samples of healthy individuals. b) Images of typical colonies of *P. aeruginosa* Tongji. c) Cortisol incubated with *P. aeruginosa* Tongji. d) C57BL/6 mice were pre‐treated with antibiotics (ABX) for 7 days, followed by *P. aeruginosa* Tongji gavage (once per day for three successive days every week for 4 weeks) and cortisol treatment for 28 days. Piperacillin was gavaged every day beginning on day 14 for 14 days. Behavioral experiments began on day 28; cortisol levels were measured on day 35. e) Serum cortisol levels in mice treated with cortisol (cortisol group), mice gavaged with *P. aeruginosa* Tongji (*P. aeruginosa* group), and mice gavaged with *P. aeruginosa* Tongji followed by piperacillin administration (piperacillin group). f) Behavioral experiments. Data are representative of at least three independent experiments, *n* = 10 mice per group. Data are mean ± S.D. One‐way ANOVA followed by post hoc Tukey multiple comparison test.

To investigate which bacteria in the intestine can degrade cortisol, we enriched the gut microbiota from healthy volunteers using a single‐carbon source culture medium, as previously described (**Figure** **2**a).^[^ ^16^ ^]^ We isolated a strain of bacteria that was identified by matrix‐assisted laser desorption ionization‐time of flight mass spectrometry (MALDI‐TOF MS) as *P. aeruginosa* (Figure 2b; Figure S1b, Supporting Information). We named this bacterium *P. aeruginosa* Tongji. Drug sensitivity tests showed that the bacterium was sensitive to piperacillin (Figure S1c, Supporting Information). *P. aeruginosa* colonizing the intestine plays a positive role in certain aspects of the host^[^ ^17^ ^]^ and is even considered a probiotic when added to the feed of farm animals.^[^ ^18^ ^]^ Moreover, *P. aeruginosa* can metabolize steroid sulphates.^[^ ^19^ ^]^ *P. aeruginosa* Tongji abundance was significantly related to depressive symptoms (Hamilton Depression Scale score) (Figure S1d, Supporting Information). However, there are currently no reports on the ability of this bacterium to degrade cortisol. To investigate whether *P. aeruginosa* Tongji can degrade cortisol, we incubated *P. aeruginosa* Tongji with cortisol in vitro. LC‐MS/MS showed that cortisol was degraded in a time‐dependent manner (Figure 2c), indicating *P. aeruginosa* Tongji can degrade cortisol.

To further investigate the effects of *P. aeruginosa* Tongji on mouse cortisol levels, we gavaged the mice with *P. aeruginosa* Tongji (*P. aeruginosa* group) (Figure 2d). We also treated *P. aeruginosa* Tongji‐gavaged mice with piperacillin (piperacillin group) to investigate the effects of *P. aeruginosa* Tongji eradication of cortisol in the mouse gut (Figure 2d; Figure S1e, Supporting Information). Interestingly, the cortisol levels in the *P. aeruginosa*‐gavaged group after cortisol treatment were lower than those in the cortisol group (18.93 ± 7.4 vs 39.4 ± 10.3, *p* < 0.0001) (Figure 2e). However, the cortisol levels of mice in the piperacillin group after cortisol treatment were not significantly different from those in the cortisol group (*p* > 0.05, Figure 2e). These results indicate that *P. aeruginosa* Tongji gavaged to mice can protect against an increase in serum cortisol levels, and these mice became susceptible to cortisol elevation after eradication of *P. aeruginosa* Tongji.

Behavioral experiments showed that compared to mice in the control group, mice treated with cortisol exhibited higher immobile and latency time in the FST, TST, NSFT and splash test (*p* < 0.05), indicating depressive‐like behavior (Figure 2f). Interestingly, mice in the *P. aeruginosa* group did not exhibit depressive‐like behavior after, whereas those in the piperacillin group did (Figure 2f). These results indicate that mice gavaged with *P. aeruginosa* Tongji were protected against cortisol‐induced depressive‐like behavior but became susceptible to depressive behavior after eradication of *P. aeruginosa* Tongji.

*P. aeruginosa* can be isolated from the faeces of ≈10% of healthy individuals.^[^ ^20^ ^]^ To further characterize the distribution of *P. aeruginosa* Tongji in the human intestinal microbiota, we analyzed the GMrepo database of 358 intestinal bacterial samples from patients with depression and 16282 samples from healthy individuals from different countries.^[^ ^21^ ^]^ Among the 358 patients with depression, *P. aeruginosa* was found in only 2 (0.56%), whereas in 16 282 healthy participants, *P. aeruginosa* was found in 1049 (6.44%; Figure S1f and Table S1, Supporting Information). Furthermore, the mean relative abundance of *P. aeruginosa* was 0.09% in patients with depression, which was lower than that in healthy individuals (0.48%; Figure S1g and Table S1, Supporting Information). These results show that the prevalence and abundance of *P. aeruginosa* in patients with depression were lower than those in healthy individuals.

We also employed small intestine contents to investigate the colonization of *P. aeruginosa* in the small intestine using 16S rRNA sequencing. The results showed that *P. aeruginosa* was detectable in control and *P. aeruginosa* gavaged mice (Figure S1h, Supporting Information). The abundance of *P. aeruginosa* in *P. aeruginosa* gavaged mice was higher than that in control mice (Figure S1h, Supporting Information). These results are consistent with previous studies,^[^ ^22^, ^23^ ^]^ indicating the colonization of *P. aeruginosa* in the small intestine.

### 2.4 A Des‐Like Enzyme from *P. aeruginosa* Tongji Degrades Cortisol in Vitro

**Figure 3:**
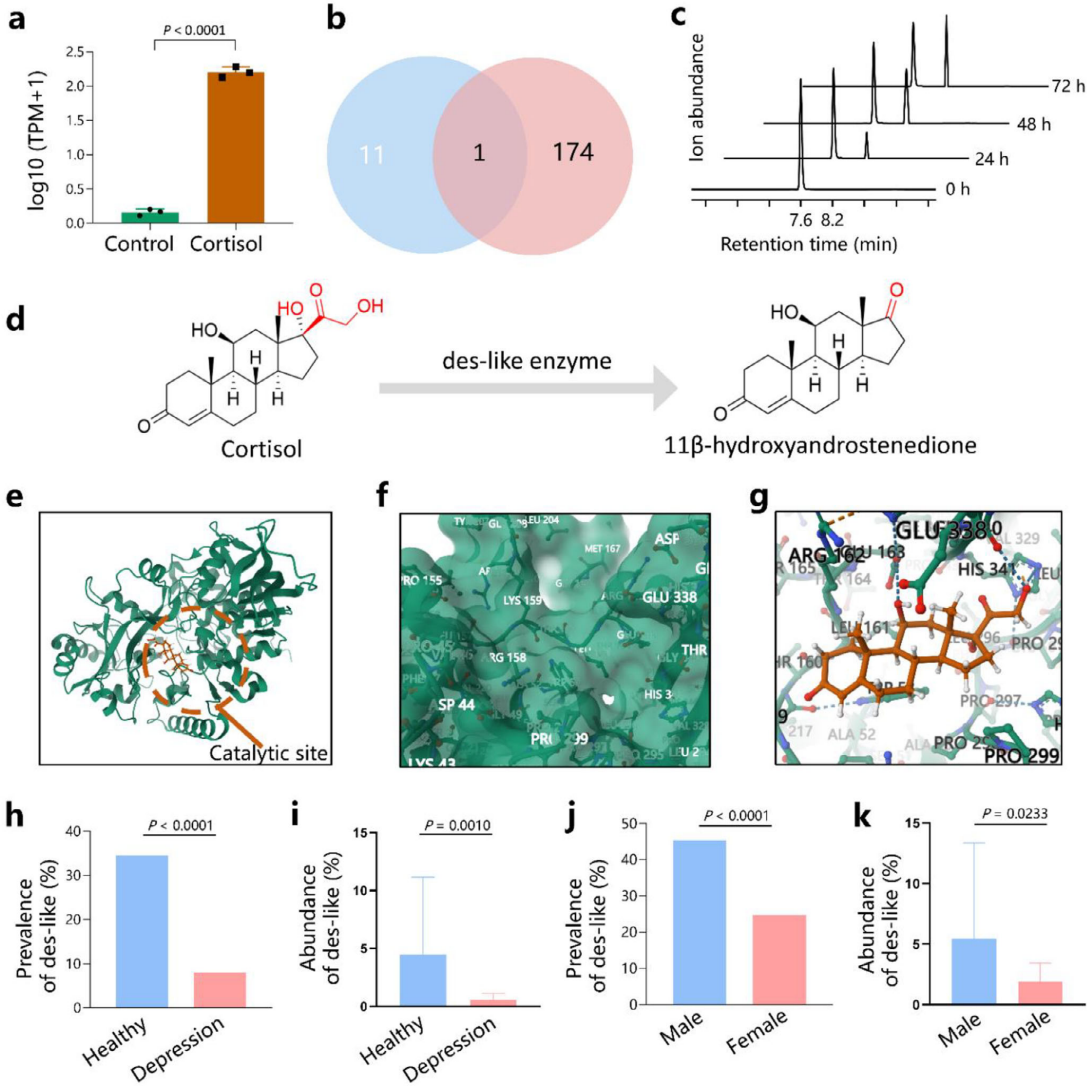
Discovery of cortisol‐degrading enzyme in *P. aeruginosa* Tongji. a) Transcriptome sequencing identified the gene VMR32_13975, which is related to steroid metabolism and is upregulated when *P. aeruginosa* Tongji is incubated with cortisol. b) The numbers in the blue and red pie charts represent the candidate proteins obtained by annotations of the Swiss‐Prot database and the comparative transcriptomic analysis (≥10‐fold upregulation in gene expression compared with cortisol). c) Experimentally determined cortisol degradation function of the des‐like enzyme. d) des‐like enzyme converts cortisol into 11β‐hydroxyandrostenedione. e) Animated representation of a des‐like enzyme with its catalytic site highlighted. f) Catalytic pockets of des‐like enzyme. g) Residue views of the pattern of cortisol binding with des‐like enzyme at the catalytic site. h) Prevalence rates of des‐like enzyme in healthy individuals and patients with depression. i) Abundance of des‐like enzyme in healthy individuals and patients with depression. j) Prevalence rates of des‐like enzyme in healthy men and women. k) Abundance of des‐like enzyme in healthy men and women. Data are representative of at least 3 independent experiments. Data are mean ± S.D. Pearson's chi‐squared test and Two‐tailed Student's test.

To investigate the mechanisms underlying cortisol degradation, we performed whole‐genome sequencing of *P. aeruginosa* Tongji (Figure S2a,b and Table S2, Supporting Information). In annotations of the Swiss‐Prot database, 11 genes in the genome of *P. aeruginosa* Tongji were potentially related to steroid metabolism (Figure S2c, Supporting Information). To investigate the genes that play a major role in cortisol degradation, we incubated *P. aeruginosa* Tongji with cortisol and performed transcriptome sequencing, using *P. aeruginosa* Tongji without cortisol incubation as the control group. Principal component analysis (PCA) of the transcriptome indicated that the gene expressions were different between *P. aeruginosa* Tongji incubated with or without cortisol (Figure S2d, Supporting Information). A total of 174 genes were upregulated >10‐fold in *P. aeruginosa* Tongji incubated with cortisol (Figure S2e, Supporting Information), suggesting that these genes may be involved in cortisol metabolism. Among the 11 genes related to steroid metabolism and 174 genes upregulated >10‐fold, one gene (expressed by the VMR32_13975 gene) was found in both cases (**Figure** **3**a,b; Table S3, Supporting Information). To experimentally determine the cortisol‐degrading function of the VMR32_13975 gene, we transformed *Escherichia coli* BL21(DE3) with pET28a containing VMR32_13975 (*E. coli*‐13975) (Figure S3a, Supporting Information) with an empty vector as a control (*E. coli‐*vector) Figure S3b, Supporting Information). After sequence verification (Table S4, Supporting Information), *E. coli*‐13975 was incubated with cortisol. LC‐MS/MS showed that cortisol was degraded in a time‐dependent manner by *E. coli*‐13975 (Figure 3c), whereas the level of the degradation product increased in a time‐dependent manner (Figure 3c). The product was identified as 11β‐hydroxyandrostenedione (11OHA4) by LC‐MS/MS (Figure S3c, Supporting Information), indicating that this enzyme has steroid‐17‐20‐desmolase activity. We name this protein as steroid‐17‐20‐desmolase‐like (des‐like) enzyme. *P. aeruginosa* abundance was significantly related to des‐like enzyme abundance (*r* = 0.692, Figure S3d, Supporting Information). No evident degradation was observed when the *E. coli* vector was incubated with cortisol (data not shown). These results showed that des‐like enzyme expressed by the VMR32_13975 gene can convert cortisol into 11OHA4 (Figure 3d).

To investigate the structure of des‐like enzyme, we modelled its 3D structure using the crystal structures of homologous proteins. The homology modelling results suggest that the arrangement of residues in the catalytic site of des‐like enzyme forms pockets (Figure 3e,f). Molecular docking simulations suggest that cortisol forms multiple hydrogen bonds with the catalytic residues and nearby residues, resulting in a binding energy of −8.233 kcal mol^−1^ (Figure 3g), suggesting that the binding between cortisol and des‐like enzyme is stable.

### 2.5 Distribution and Abundance of the Des‐Like Protein in the Gut Microbiota

We explored the distribution of des‐like enzymes in microbial communities using the des‐like enzyme sequence as a query. The BLASTp search identified 1038 putative des‐like proteins from four microbial species: *P. aeruginosa*, *P. fluorescens*, *Acinetobacter baumannii*, and *Enterobacter cloacae* (Table S5, Supporting Information). We next reanalyzed the abovementioned 358 samples from patients with depression and 16282 samples from healthy individuals. The prevalence rates of *P. aeruginosa* (6.44% vs 0.56%), *P. fluorescens* (6.31% vs 0.84%), *A. baumannii* (3.39% vs 0.28%), and *E. cloacae* (5.87% vs 0%) were higher in the healthy group than in the depression group (Figure S3e and Table S1, Supporting Information). Moreover, the abundances of *P. aeruginosa* (0.48% vs 0.001%), *P. fluorescens* (0.07% vs 0.002%), *A. baumannii* (0.11% vs 0.003%), and *E. cloacae* (1.00% vs 0%) were higher in the healthy group than in the depression group (Figure S3f and Table S1, Supporting Information).

To further investigate the distribution of des‐like enzyme in the human gut, we used protein‐coding sequences of the des‐like enzyme gene as queries to search for the most similar nucleotide fragments in *P. aeruginosa*, *P. fluorescens* and *A. baumannii* genomes. Primers were designed based on the nucleotide fragments of each bacterium. Multiple qPCR was performed on DNA isolated from faecal samples of healthy individuals (*n* = 139) and patients with depression (*n* = 100) to explore the distribution of des‐like enzyme. The results showed that the prevalence rates of des‐like enzyme were higher in healthy individuals than in patients with depression (34.53% vs 8.00%, *p* < 0.0001) (Figure 3h). Moreover, the abundance of des‐like enzyme was higher in healthy individuals than in patients with depression (4.48 ± 6.67 vs 0.54 ± 0.56, *p* = 0.001) (Figure 3i). These results suggest that des‐like enzyme is more widespread in the gut microbiota of healthy individuals than in those of patients with depression.

Since the incidence of depression in women is twice that of men,^[^ ^24^ ^]^ we further investigated whether there is a sex difference in the distribution of des‐like enzyme in healthy individuals. The results showed that the prevalence rate of des‐like enzyme was higher in healthy men than in healthy women (45.45% vs 24.66%, *p* < 0.0001) (Figure 3j). The abundance of des‐like enzyme in healthy men was also higher than in healthy women (5.46 ± 7.92 vs 1.88 ± 1.55, *p* = 0.0233) (Figure 3k). These results showed that des‐like enzyme is more widespread in the gut microbiota of healthy men than women, suggesting that the sex difference in enzyme distribution may partially explain why women are more prone to depression than men.

To explore the discriminative ability of des‐like enzyme abundance in individuals susceptible to depression, the receiver operating characteristic (ROC) curve was determined between healthy individuals and patients with depression. The area under the curve is 0.857 (sensitivity = 87.5%, specificity = 75.0%) (Figure S3g and Table S6, Supporting Information). The area under the ROC in the independent validation cohort (*n* = 56) is 0.784 (Figure S3h, Supporting Information), indicating that des‐like enzyme has good discriminative ability in individuals susceptible to depression. These results suggest that des‐like enzyme may be a potential biomarker for assessing susceptibility to depression.

### 2.6 The Des‐Like Protein Derived from Gut Bacteria Protects against Cortisol‐Induced Depressive‐Like Behavior and Brain Inflammation

**Figure 4:**
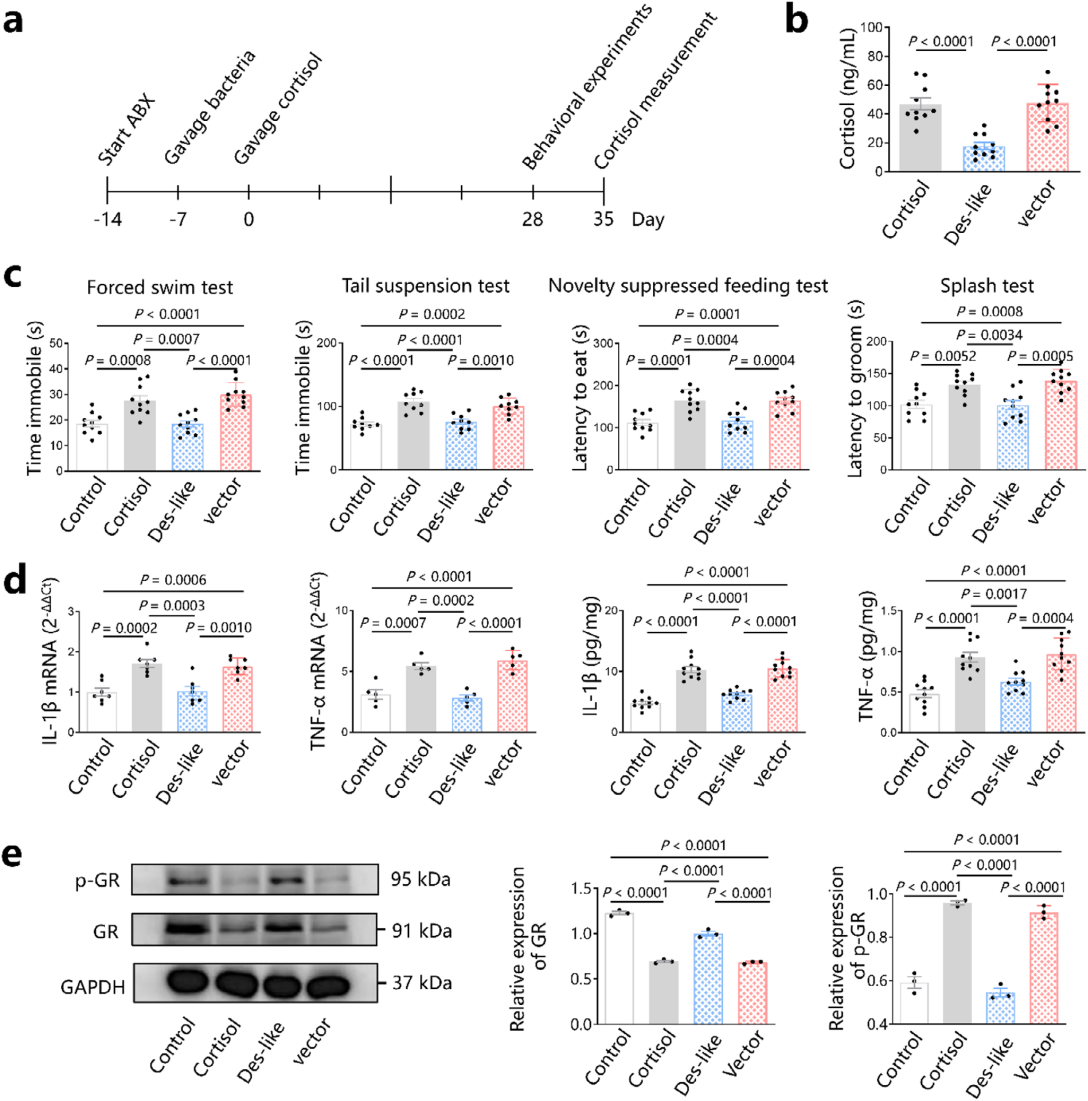
*E. coli* heterologous expression of des‐like enzyme protects against increases in cortisol levels and depression. a) C57BL/6 mice were pre‐treated with antibiotics (ABX) for 7 days, followed by *E. coli*‐13975 (heterologous expression of des‐like enzyme) or *E. coli*‐vector gavage (once per day for three successive days every week for 4 weeks) and cortisol treatment for 28 days. Behavioral experiments began on day 28; cortisol levels were measured on day 35. b) Serum cortisol levels in mice treated with cortisol (cortisol group), mice gavaged with *E. coli*‐13975 (des‐like group), and mice gavaged with *E. coli*‐vector (vector group). c) Behavioral experiments. d) Detection of IL‐1β and TNF‐α mRNA and protein in hippocampal samples. e) Detection of GR and p‐GR using western blotting (WB). GR: glucocorticoid receptor. p‐GR: glucocorticoid receptor phosphorylation protein. Data are representative of at least three independent experiments, *n* = 3–10 mice per group. Data are mean ± S.D. One‐way ANOVA followed by post hoc Tukey multiple comparison test.

To investigate whether gut bacteria expressing des‐like enzyme can resist elevated cortisol levels, we gavaged mice with *E. coli*‐13975 (des‐like group), or with *E. coli*‐vector gavaged (vector group) as a control (**Figure** **4**a; Figure S3i, Supporting Information). LC‐MS/MS showed that the serum cortisol levels of mice in the des‐like group were lower than those in the cortisol group (17.7 ± 7.9 vs 46.9 ±13.3, *p* < 0.0001) (Figure 4b). However, the serum cortisol levels of mice in the vector group were higher than those in the des‐like enzyme groups (47.5 ± 12.8 vs 17.7 ± 7.9, *p* < 0.0001), and no significant difference was observed compared with those in the cortisol group (47.5 ± 12.8 vs 46.9 ±13.3, *p* > 0.05) (Figure 4b). These results suggest that gut bacteria expressing the des‐like enzyme protect against increased cortisol levels. Mice were susceptible to cortisol elevation when the gut bacteria did not express the des‐like protein.

Behavioral experiments showed that after cortisol treatment, immobile and latency time in the FST, TST, NSFT and splash test were not significant different in the des‐like enzyme group compared to those in the control group, indicating no depressive‐like behavior in des‐like enzyme group (*p* > 0.05, Figure 4c). However, mice in the vector group exhibited depressive‐like behavior after cortisol treatment without a significant difference from those in the cortisol group, in contrast to mice in the control and des‐like enzyme groups (*p* < 0.05, Figure 4c). These results indicate that gut bacteria expressing des‐like enzyme improve cortisol‐induced depressive‐like behavior. Mice were susceptible to cortisol and tended to develop depressive‐like behavior when the gut bacteria did not express the des‐like protein.

Cortisol‐induced depression is associated with elevated levels of the proinflammatory factors interleukin (IL)‐1β and tumour necrosis factor (TNF)‐α in the hippocampus.^[^ ^4^ ^]^ To investigate whether gut bacteria expressing des‐like enzyme could protect against the cortisol‐induced elevation of IL‐1β and TNF‐α in the hippocampus, we detected the expression levels of these cytokines in murine hippocampi. The results of reverse transcription PCR showed that mice in the cortisol group had higher mRNA levels of IL‐1β (*p* = 0.0002) and TNF‐α (*p* = 0.0007) in the hippocampus than mice in the control group (Figure 4d). However, the mRNA expression levels of IL‐1β (*p* = 0.0003) and TNF‐α (*p* = 0.0002) in mice of the des‐like enzyme group were significantly lower than those of the cortisol group, with no significant difference from those of the control group (*p* > 0.05, Figure 4d). The mRNA expression levels of IL‐1β and TNF‐α in vector group mice were significantly higher than those in control and des‐like enzyme group mice (*p* < 0.05, Figure 4d). The results of IL‐1β and TNF‐α protein levels were similar to the corresponding mRNA levels (Figure 4d). These results indicate that gut microbiota expressing the des‐like enzyme protect against these cortisol‐induced proinflammatory factors in the hippocampus.

Since cortisol‐induced increases in proinflammatory factors in the hippocampus are related to decreases in glucocorticoid receptors (GR),^[^ ^25^ ^]^ we next explored whether bacteria expressing des‐like enzyme in the gut can protect against decreased glucocorticoid receptor expression in the hippocampus. Western blot analysis indicated a decrease in the expression levels of glucocorticoid receptors (*p* < 0.0001) and glucocorticoid receptor phosphorylation (p‐GR) (*p* < 0.0001) in the hippocampus of mice in the cortisol group (Figure 4e). Interestingly, mice in the des‐like enzyme group showed increased expression of GR and p‐GR in the hippocampus compared to those in the cortisol group (*p* < 0.0001) and exhibited a non‐significant difference compared with those in the control group (*p* > 0.05, Figure 4e). However, mice in the vector group exhibited decreased expression of GR and p‐GR compared with that of the control and des‐like groups (Figure 4e). These results indicate that bacteria in the gut with the ability to express des‐like enzyme protect against cortisol‐induced decreases in glucocorticoid receptor expression in the hippocampus.

### 2.7 The 11OHA4 Produced by Des‐Like Enzyme Indirectly Improves Depressive‐Like Behavior

**Figure 5:**
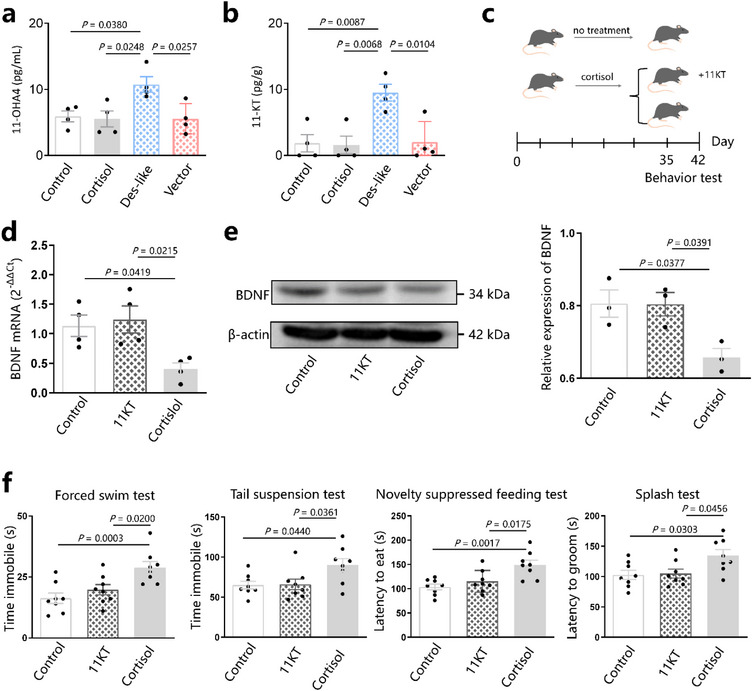
The 11OHA4 indirectly improves depressive‐like behavior. a) Detection of serum 11‐OHA4 in mice. b) Detection of brain 11‐KT in mice. c) Mice in the 11KT group were treated with 11KT for 35 days, followed by cortisol treatment for 28 days. Mice in the cortisol group were treated with cortisol for 28 days. Behavioral experiments began on day 28; cortisol levels were measured on day 35. d) Detection of BDNF mRNA and protein in hippocampal samples. e) Representative Western blots analyzing BDNF protein expression in the brain of mice. f) Behavioral experiments. 11OHA4: 11β‐hydroxyandrostenedione. 11‐KT: 11‐ketotestosterone. Data are representative of at least three independent experiments, *n* = 3–10 mice per group. Data are mean ± S.D. One‐way ANOVA followed by post hoc Tukey multiple comparison test.

The 11OHA4 is an 11‐oxygenated androgen without detectable androgen activity.^[^ ^26^ ^]^ By detecting 11OHA4 in serum, we found that 11OHA4 levels in healthy individuals were higher than those in patients with depression (*p* < 0.0001, Figure S3j, Supporting Information). We also found that 11OHA4 levels in des‐like groups were higher than those in the vector group and control group (10.75 ± 2.34 vs 5.90 ± 1.63 pg mL^−1^, *p* = 0.0380) (**Figure** **5**a), indicating that des‐like enzyme expressed by gut microbe increased 11OHA4 in circulation. The 11OHA4 is metabolized in tissues to 11‐ketotestosterone (11KT). The 11‐KT is a bioactive androgen with a potency similar to that of testosterone,^[^ ^27^ ^],^ which may improve depressive‐like behavior via increasing brain‐derived neurotrophic factor (BDNF) in the hippocampus.^[^ ^28^ ^]^ We incubated brain tissue homogenate with 11OHA4 and detected the 11KT. The results showed that the concentration of the 11KT increases in a time‐dependent manner (Figure S3k, Supporting Information). Next, we detected 11KT in mice brain and found that 11KT in des‐like groups was higher than that in vector group (9.50 ± 2.53 vs 2.05 ± 3.09 pg g^−1^, *p* = 0.0104) and control group (9.50 ± 2.53 vs 1.85 ± 2.61 pg g^−1^, *p* = 0.0087), indicating that des‐like enzyme expressed by gut microbe increased 11KT in mice brain (Figure 5b). We next gavaged cortisol‐treated mice with 11KT to explore the effect of 11KT on depressive‐like behavior (Figure 5c). The results showed that BDNF mRNA and protein were lower in cortisol‐treated mice than in control mice (Figure 5d,e). These results are similar to the findings of previous research,^[^ ^29^ ^]^ suggesting that 11KT improved the cortisol‐induced decrease in BDNF mRNA and protein. Behavioral experiments showed that 11KT improved cortisol‐induced depressive‐like behavior (Figure 5f). These results suggest that the 11OHA4 produced by des‐like enzyme indirectly improved depressive‐like behavior via increasing BDNF in the hippocampus.

### 2.8 The Effect of *ΔDes‐Like* on Cortisol and Depressive‐Like Behavior in Mice

To explore whether the observed antidepressant effect is due to the direct metabolism of cortisol by *P. aeruginosa* Tongji, we conducted a *des‐like* knockout strain *P. aeruginosa* Δ*des*. The *P. aeruginosa* Δ*des* cannot efficiently metabolize cortisol in vitro (Figure S4a, Supporting Information). After *P. aeruginosa* Δ*des* was gavaged to mice, the cortisol levels were not significantly different compared to cortisol‐treated mice (Figure S4b, Supporting Information). Behavior tests showed that *P. aeruginosa* Δ*des* did not improve cortisol‐induced depressive‐like behavior (Figure S4c, Supporting Information). These results indicated that the observed antidepressant effect is due to the direct metabolism of cortisol by *P. aeruginosa* Tongji.

### 2.9 Construction of Probiotics Expressing the Des‐Like Enzyme

**Figure 6:**
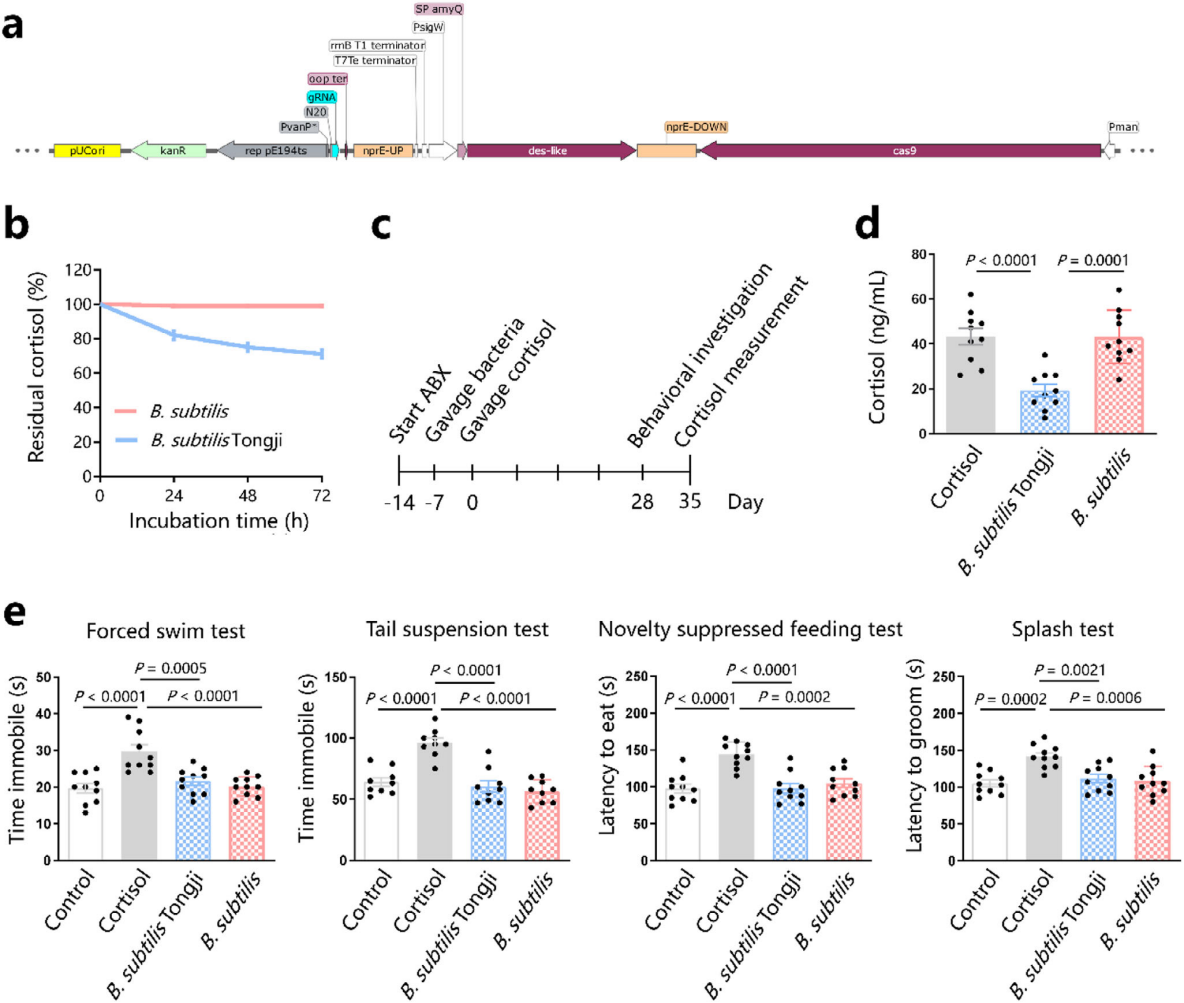
Generation of the probiotic *B. subtilis* Tongji expressing the des‐like enzyme. a) Introduce the gene encoding the des‐like protein into the genome of *B. subtilis* 168 to construct *B. subtilis* Tongji. b) Cortisol levels of *B. subtilis* Tongji or *B. subtilis* incubated with cortisol in vitro. c) C57Bl/6 mice were pre‐treated with antibiotics (ABX) for 7 days, followed by *B. subtilis* Tongji or *B. subtilis* gavage (once per day for three successive days every week for 4 weeks) and cortisol treatment for 28 days. Behavioral experiments began on day 28; cortisol levels were measured on day 35. d) Serum cortisol levels in mice treated with cortisol (cortisol group), mice gavaged with *B. subtilis* Tongji (*B. subtilis* Tongji group), and mice gavaged with *B. subtilis* (*B. subtilis* group). e) Behavioral experiments. Data are representative of at least three independent experiments, *n* = 10 mice per group. Data are mean ± S.D. One‐way ANOVA followed by post hoc Tukey multiple comparison test.

The protective effect of des‐like enzyme in the intestine against cortisol elevation and depressive‐like behavior suggests that bacteria expressing des‐like enzyme could be used as an intervention measure to prevent or improve depression. In our experiments, *P. aeruginosa* Tongji and *E. coli*‐13975 protected against increased cortisol levels and depressive‐like behavior in mice, but these bacteria can cause human infectious diseases.^[^ ^30^, ^31^ ^]^ Therefore, we did not regard *P. aeruginosa* Tongji and *E. coli*‐13975 as competent for preventing or improving depression and considered constructing a probiotic that can express des‐like enzyme. Although an increasing number of clinical trials have found that probiotics can improve depressive symptoms in patients, current evidence suggests that probiotics cannot reduce or prevent high levels of cortisol.^[^ ^32^ ^]^ Considering that high cortisol levels increase the risk of relapse in patients with depression whose symptoms have been relieved,^[^ ^33^ ^]^ the development of a probiotic that can lower cortisol levels may help prevent and improve depression. To this end, we inserted the gene encoding des‐like enzyme into the genome of *Bacillus subtilis* (**Figure** **6**a), known to improve depression,^[^ ^34^ ^]^ to create *B. subtilis* Tongji, thereby allowing it to constitutively express des‐like enzyme. After sequence verification, *B. subtilis* Tongji was incubated with cortisol (1 g L^−1^) in vitro. LC‐MS/MS showed that cortisol was degraded in a time‐dependent manner when incubated with *B. subtilis* Tongji, whereas no relevant degradation was observed when cortisol was incubated with *B. subtilis* (Figure 6b).

To explore the protective effect of *B. subtilis* Tongji on elevated cortisol levels and depressive‐like behavior, mice were gavaged with *B. subtilis* Tongji or *B. subtilis* (Figure 6c). Although behavioral experiments showed that both *B. subtilis* Tongji and *B. subtilis* gavage protected against cortisol‐induced depressive‐like behavior, LC‐MS/MS showed that cortisol levels in mice gavaged with *B. subtilis* Tongji were lower than those in the cortisol group (19.2 ± 8.5 vs 43.2 ± 11.6 ng mL^−1^, *p* < 0.0001), whereas cortisol levels did not significantly differ between mice gavaged with *B. subtilis* compared to those in the cortisol group (43.0 ± 11.9 vs 43.2 ± 11.6 ng mL^−1^, *p* > 0.05) (Figure 6d,e). These results suggest that *B. subtilis* does not improve cortisol susceptibility in the host, and *B. subtilis* Tongji is superior to *B. subtilis* in protecting against stress.

## 3 Discussion

Long‐term or severe life stress can induce or exacerbate depression. For example, work pressure, interpersonal problems, economic difficulties, unemployment, heartbreaks, and other life events can trigger depression. Such stress activates the HPA axis, and the stress hormone cortisol is the main effector of this stress response system.^[^ ^3^ ^]^ Chronic exposure to cortisol can lead to the desensitization of glucocorticoid receptors and the absence of an appropriate response to cortisol.^[^ ^25^ ^]^ Consequently, sustained, non‐resolving inflammation is promoted in the central nervous system, resulting in symptoms of depression.^[^ ^4^ ^]^ Although the impact of stress on depression is widespread, considerable differences exist among individuals. Some individuals exhibit strong resilience, whereas others are more susceptible to the effects of stress and develop depression. Our findings suggest that the cortisol levels are related to the ability of the gut microbiota to degrade cortisol. Bacterial des‐like enzyme may regulate cortisol and improve depression by converting cortisol into androgen.

Chronic treatment with antibiotics in mice resulted in increased stress hormone levels at baseline^[^ ^35^ ^]^ and after social exposure.^[^ ^8^ ^]^ However, the mechanism by which the gut microbiota of rodents reduces stress responses and whether the human gut microbiota induces similar resistance to stress remains unclear. Our results show that the gut microbiota from healthy individuals, but not from patients with depression, protected mice against stress‐induced cortisol elevation. Moreover, intestinal microbiota with varying abilities of cortisol degradation offered varying degrees of protection against cortisol elevation. Mice with gut microbiota lacking the ability to degrade cortisol were susceptible to cortisol elevation and depressive‐like behavior, suggesting that the protective ability of the gut microbiota against stress depends on the ability to degrade cortisol. Individual differences in the ability of the gut microbiota to degrade cortisol can partially explain individual differences in stress susceptibility.

Cortisol is mainly synthesized by the adrenal glands and secreted into the peripheral blood. Cortisol in the blood circulates through the enterohepatic axis and enters the intestine, where it comes into contact with gut microbes and is subsequently reabsorbed into the bloodstream.^[^ ^36^ ^]^ Moreover, one study has shown that the ileum may produce cortisol. Thus, cortisol from the ileum and adrenal glands comprises the circulating cortisol pool.^[^ ^35^ ^]^ During this process, the gut microbiota may affect the cortisol levels through metabolism. Researchers have discovered several bacterial species that can degrade cortisol. For example, *Streptomyces roseochromogenes* can metabolize hydrocortisone into 16α‐hydroxy hydrocortisone.^[^ ^37^ ^]^ *Propionibacterium* spp. degrade cortisol via the kstD enzyme.^[^ ^38^ ^]^ However, these studies focused on the production of drugs or drug intermediates through the metabolic capacity of bacteria. Currently, a few work reports exist on the presence of cortisol‐degrading bacteria in the human gut and on whether the ability of gut bacteria to degrade cortisol affects human health. A recent descriptive study found that *Ruminococcus gnavus* isolated from patients with Cushing syndrome can degrade cortisol in vitro.^[^ ^39^ ^]^ However, it remains unknown whether this bacterium affects humans via its cortisol‐degrading ability. In the present study, we isolated *P. aeruginosa* Tongji, which can degrade cortisol, from the faecal microbiota of healthy individuals. The abundance of *P. aeruginosa* was significantly reduced in individuals with depression. The reduced abundance of *P. aeruginosa* may be partly attributed to diet. Diet constitutes a pivotal determinant of gut bacterial assembly and genes.^[^ ^40^ ^]^ Individuals with depression or pre‐clinical depression had different food preferences.^[^ ^41^ ^]^ The abundance of des‐like enzyme was significantly reduced in females. It has been reported that the composition of the gut microbiota of male mice was distinct from that of females at the time of puberty. Another study revealed the strong impact of gonadectomy and hormone replacement on the composition of microbiota in mice.^[^ ^42^ ^]^ Therefore, the changed abundance of des‐like enzyme abundance may be partly attributed to sex hormones.

*P. aeruginosa* Tongji‐gavaged mice exhibited resistance to cortisol elevation and depressive‐like behaviors. Eradication of *P. aeruginosa* Tongji with antibiotics abolished these protective effects. These results suggest that cortisol‐degrading bacteria in the gut protect the host against stress. The prevalence rates and abundances of *P. aeruginosa* Tongji were higher in healthy individuals than in patients with depression. Furthermore, since *S. roseochromogenes, Propionibacterium* spp., and *R. gnavus* were found to degrade cortisol, we explored the prevalence and abundance of these strains in the GMrepo database and found the abundances of *Propionibacterium* spp. and *R. gnavus* were decreased in patients with depression, whereas *S. roseochromogenes* was not detected in the human gut (Tables S1 and S7, Supporting Information).

Previous studies have found that some bacteria in nature can convert steroids into androgens.^[^ ^43^, ^44^, ^45^, ^46^, ^47^, ^48^, ^49^ ^]^ *Clostridium scindens*, *Butyricicoccus desmolans*, *Clostridium cadavaris*, and *Propionimicrobium lymphophilum* have been reported to convert cortisol to 11OHA4. However, *C. scindens*, *B. desmolans* and *C. cadavaris* was not found in the human gut (median relative abundances <0.01%), and *P. lymphophilum* was found in only 0.032% (12/37500) of humans with low relative abundances (0.01–0.05%) according to the GMrepo database, indicating that these bacteria may not be clinically significant. In the present study, we identified a des‐like enzyme in *P. aeruginosa* Tongji. Gut bacteria that express des‐like enzyme protect the host against stress by degrading cortisol, suggesting that the des‐like enzyme gene expressed by gut microbiota is a potential biomarker for evaluating psychological endurance. Testosterone and estradiol, along with cortisol, belong to steroid hormones. Testosterone and estradiol are also associated with depression. Therefore, we also investigated the relationship between enzymes and testosterone and estradiol, and the results showed that enzymes cannot metabolize testosterone and estradiol (data not shown). Structurally, testosterone and estradiol do not have side chains, while des‐like enzymes mainly act on the side chains, so testosterone and estradiol are not substrates of des‐like enzymes. Interestingly, the 11OHA4 produced by des‐like enzyme belongs to the family of 11‐oxygenated androgen,^[^ ^27^ ^]^which can indirectly activate androgen receptors,^[^ ^50^ ^]^ and improve depressive‐like behavior.^[^ ^28^ ^]^ By analyzing the distribution of des‐like enzyme, our results showed that des‐like enzyme is more widespread in the gut microbiota of healthy men than women, suggesting that the sex difference in enzyme distribution may partially explain why women are more prone to depression than men. Our results showed the colonization of *P. aeruginosa* in mice small intestine. As cortisol is a lipid‐soluble molecule, oral gavage administration in mice results in its substantial absorption in the small intestine, followed by entry into the portal vein system and subsequent hepatic metabolism. The colonization of *P. aeruginosa* in the small intestine decreases the absorption of cortisol.

We identified four additional bacterial species containing des‐like enzymes: *P. aeruginosa*, *P. fluorescens*, *A. baumannii*, and *E. cloacae*. These strains may not necessarily have antidepressant effects. For example, *E. cloacae* lead to depressive symptoms via producing ammonia.^[^ ^51^ ^]^ Compared to healthy individuals, *A. baumannii* ^[^ ^52^ ^]^ is higher in patients with depression. Considering that these bacteria are not probiotics, it is reasonable that they may have a dual effect on depression. That's why we introduce des‐like genes into probiotics *B. subtilis*, so we can obtain a probiotic that expresses des‐like genes with minimal side effects. By inserting the gene encoding the des‐like enzyme into the genome of the probiotic *B. subtilis* 168, we obtained *B. subtilis* Tongji, which constitutively expresses the des‐like enzyme. This strain protected the murine host against cortisol elevation and depressive‐like behavior. *B. subtilis* is a safe probiotic that has been commercialized, whereas *P. aeruginosa* can cause various infectious diseases such as respiratory tract infections,^[^ ^53^ ^]^ urinary tract infections,^[^ ^54^ ^]^ wound infections,^[^ ^55^ ^]^ and blood infections.^[^ ^56^ ^]^ Evidence supports antidepressant roles for testosterone in both men and women.^[^ ^57^ ^]^ Therefore, *B. subtilis* Tongji is superior to traditional probiotics in its protection against cortisol elevation. High cortisol levels increase the risk of relapse in patients with depression whose symptoms have been relieved, and the normalization of cortisol levels is an important prerequisite for clinical improvement.^[^ ^33^ ^]^ *B. subtilis* Tongji may be superior to traditional probiotics in preventing relapse of depression. However, these speculations require confirmation by clinical trials.

Our study has some limitations. The low abundance of *P. aeruginosa* is only part of the cause of the reduced expression of des‐like enzymes in the gut, as there are other bacterial species harboring des‐like enzymes. This may lead to *P. aeruginosa* not being the key contributor.

## 4 Conclusion

Our study found a link between gut microbiota and cortisol, suggesting that the occurrence of depression is related to the reduced ability of gut microbiota to degrade cortisol. Furthermore, we elucidated the mechanism by which the gut microbiota degrades cortisol and constructed a probiotic that can degrade cortisol. This probiotic is a promising intervention to combat stress‐induced disorders. These conclusions should be verified by clinical trials or prospective cohort studies.

## 5 Experimental Section

### Human Participants

All protocols were approved by the Ethics Committee (approval no. WDRY2020‐K191). Samples were collected with informed consent from all participants or their legal guardians. The study participants were recruited from Renmin Hospital of Wuhan University. Patients (20–60 years old) in the depression group met the DSM‐V diagnostic criteria for depression. The study excluded patients with hypercortisol syndrome, dementia, substance dependence, pituitary diseases or endocrine disorders that may affect cortisol levels and a history of taking antidepressants in the past three months. Age‐matched healthy volunteers who did not meet the DSM‐V criteria for depression were also enrolled. Routine blood, kidney, and liver function tests; chest radiography; urine and faeces evaluations; and physical examinations were performed, and the clinical indices were all within normal limits. The exclusion criteria included metabolic syndrome, viral infections, kidney diseases, hypertension, cancer, inflammatory bowel disease, celiac disease, diabetes mellitus, and endocrine disorders. Patients or the public were not involved in the design, conduct, reporting, or dissemination plans of the research

### Mice

Six‐week‐old C57BL/6 male mice were purchased from GemPharmatech Co., Ltd. and housed at the School of Basic Medicine, Tongji Medical College. The mice were fed a conventional diet and maintained at 21–24 °C and 40–70% humidity with lights on throughout the day. The mice were randomly assigned to age‐ and sex‐matched treatment groups. All samples were collected between 8.00 a.m. and 12.00 a.m. to minimize circadian fluctuations in biological measurements. Mice were gavaged with 20 mg kg^−1^ cortisol (Aladdin, China) once daily for 28 consecutive days to investigate the effect of gut microbes on cortisol. Studies were carried out in accordance with the guidelines of the Institutional Animal Care and Use Committee. Mice were administered cortisol to establish a cortisol‐induced depression model. The method of supplementing cortisol to establish a depression model was established by a previous study.^[^ ^14^, ^15^ ^]^ Mice reach their experimental endpoint when bacterial gavage was completed.

### Gut Microbiota Transplantation

Transplantations of gut microbiota were performed in accordance with a transplantation protocol described previously.^[^ ^58^ ^]^ Details of the faecal microbiota transplants are listed in Table S8 (Supporting Information).

### Antibiotic Treatment

To deplete the gut microbiota, mice were supplied with drinking water sweetened with 1% w/v of sucrose containing ampicillin (1 g L^−1^), vancomycin (0.5 g L^−1^), metronidazole (0.5 g L^−1^), and neomycin (1 g L^−1^) and filtered with a 0.22 µm filter.

### Mass Spectrometry Analysis

Cortisol concentrations were measured using LC‐MS/MS. Commercial cortisol and 11OHA4 standards were purchased from Aladdin. Working solutions were obtained by diluting the stock solution to 0.01 mg L^−1^. The cortisol in the sample was extracted using liquid‐liquid extraction, which was performed by adding ethyl acetate at three times the sample volume and vortexing for 1 min, followed by centrifugation for 10 min at 10000 × *g*. The analysis was performed using a Dionex Ultimate 3000 HPLC system coupled with a Q‐Exactive Orbitrap mass spectrometer (Thermo Fisher Scientific, San Jose, CA, USA) in positive mode. Formic acid was mixed with acetonitrile to form the mobile phase (70:30, v/v). The injection volume was 5 µL and the flow rate was 0.250 mL min^−1^. ACQUITY UPLC BEH C18 Columns (Waters, Milford, MA) were used for chromatographic separation. A binary mobile phase was composed of water (solvent A) and acetonitrile (solvent B).

### Behavioral Experiments

Mice were subjected to the splash test, NSFT, TST, and FST, in that order, as previously described.^[^ ^59^ ^]^

### Enrichment of Cortisol‐Degrading Strains

Faecal samples were added to selective medium (30 mL) containing mineral salt medium and 50 mg L^−1^ cortisol. The mineral salt medium comprised 2 mm MgSO_4_·7H_2_O, 9 mm NaCl, 19 mm NH_4_Cl, 0.1 mm CaCl_2_·2H_2_O, 22 mm KH_2_PO_4_, and 48 mm Na_2_HPO_4_. The sample was incubated at 37 °C on a rotary shaker at 250 rpm. After three days, 1 mL of the cell suspension was combined with 29 mL of the selective medium. This procedure was replicated four times. Bacterial colonies were obtained by inoculating the final culture suspension onto blood agar plates.

### Bacteria Gavage

*P. aeruginosa* Tongji, *E. coli*‐13975, *E. coli*‐vector, and *B. subtilis* Tongji were grown separately in LB broth overnight at 37 °C on a rotary shaker at 250 rpm. The following day, bacteria were subcultured to the mid‐logarithmic phase (OD_600_ = 0.5), and mice were administered 1×10⁸ CFU by gavage. Bacteria were administered by oral gavage once per day for three successive days every week for 4 weeks based on previous studies.

### An Abundance of Bacteria

The taxonomic abundances in gut microbiota samples from patients with depression and healthy human participants with phenotypes D006262 and D003863, respectively, were downloaded from the GMrepo database (https://gmrepo.humangut.info/home).

### Whole‐Genome Sequencing

Genomic DNA from *P. aeruginosa* Tongji was isolated using a Bacterial Genomic DNA Extraction Kit (Tiangen, China) according to the manufacturer's protocol. Whole‐genome sequencing was performed and assembled by Shanghai Majorbio Bio‐Pharm Technology Co., Ltd. Data were analyzed using the online version of Majorbio Cloud Platform.

### Molecular Docking

AutodockVina 1.2.2 (https://autodock.scripps.edu/), an in silico protein‐ligand docking software, was used to study the binding affinities and interaction modes of cortisol with the des‐like protein.^[^ ^60^ ^]^ The molecular structure of cortisol was obtained from PubChem (https://pubchem.ncbi.nlm.nih.gov/).^[^ ^61^ ^]^ Protein PDB files were downloaded from SWISS‐MODEL (https://swissmodel.expasy.org/interactive). To conduct docking analyses, all protein and molecular files were transformed into PDBQT format. The docking scores of Autodock Vina 1.2.2 were obtained as estimations of ΔG (kcal mol^−1^).

### Heterologous Expression

The gene VMR32_13975 was introduced into the pET28a plasmid using the multicloning site 5′ NcoI–HindIII 3′. The recombinant pET28a plasmid was verified by sequencing using the T7 and T7 ter primers and introduced into *E. coli* BL21(DE3) to construct *E. coli*‐13975 (Table S9, Supporting Information). For constructing *B. subtilis* Tongji, nprE was used as the insertion site, and PsigW was used as the promoter. Optimized gene VMR32_13975 (Table S4, Supporting Information) was inserted into *B. subtilis* 168 to construct *B. subtilis* Tongji.

### Abundance of Des‐Like Enzyme

The sequence of the des‐like enzyme was used as a query in a BLASTp search of the non‐redundant protein sequence database (NR) at the National Centre for Biotechnology Information (NCBI). The taxonomic information of the resulting des‐like protein homologues (e‐value ≤1×10^−10^, coverage ≥80%, identity ≥80%) was downloaded.

To investigate the distribution of des‐like enzyme in the human gut accurately, protein sequences were used from des‐like enzyme as queries in tblastn to search for the most similar nucleotide fragments in *P. fluorescens*, *E. cloacae*, and *A. baumannii* genomes (Table S10, Supporting Information). Primers were designed using the highest‐scoring nucleotide fragment from each bacterium as a template using Primer‐Blast. The distribution and abundance of enzymes in human faecal DNA were explored using dye‐based multiplex qPCR. Due to the 100% identify of template nucleotide fragments between *P. aeruginosa* and *P. fluorescens*, a pair of primers were designed using the same template to simultaneously amplify *P. aeruginosa* and *P. fluorescens*. Therefore, a total of three pairs of primers were used in dye‐based multiplex qPCR to investigate the distribution of des‐like enzyme (Table S9, Supporting Information). The universal primer EUB was used as an internal reference (Table S8, Supporting Information). The relative expression of des‐like genes was evaluated using the 2^−ΔΔCt^ method.

### IL‐1β and TNF‐α mRNA Detection

For IL‐1β and TNF‐α mRNA detection in hippocampal tissues, qPCR analyses were performed. The primers for IL‐1β, TNF‐α, and actin are shown in Table S9 (Supporting Information). The relative expression of the targeted genes was evaluated using the 2^−ΔΔCt^ method.

### IL‐1β and TNF‐α Protein Detection

ELISA kits from Elabscience in China were employed to quantify cytokine levels of IL‐1β (sensitivity 3.2 pg mL^−1^) and TNF‐α (sensitivity 7.81 pg mL^−1^). Hippocampal homogenates were prepared according to the manufacturer's instructions. The optical density of the chemiluminescent protein signal was quantified.

### Western Blotting

Hippocampal homogenates were treated with RIPA lysis buffer (Servicebio, Wuhan, China). Following sonication and centrifugation, hippocampal proteins were electrophoresed and transferred onto a PVDF membrane (Millipore, Shanghai, China). Membranes were incubated in 5% skim milk for blocking and subsequently exposed to primary antibodies targeting glucocorticoid receptors (Affinity Bioscience, Jiangsu, China) and secondary antibodies (Proteintech, Wuhan, China). Finally, the membranes were visualized with an ECL kit from Bio‐Rad and imaged with a QuickChemi Imager (QuickChemi 5100; Monna, Jiangsu, China).

### Statistical Analysis

Experiments were independently performed at least three times. Error bars indicate the standard deviation from the mean. Using GraphPad Prism v8.0, statistical significance was evaluated using the two‐tailed Student's t‐test for two groups or one‐way ANOVA followed by a post hoc Tukey test for multiple comparison analyses. Multiple testing correction was performed.

## Conflict of Interest

Di Li have filed patents on this work. All other authors declare no competing interests.

## Supporting information



Supporting Information

Supplemental Table

## Data Availability

The genome of *P. aeruginosa Tongji* is deposited at GenBank (Accession: CP142601) and is publicly available as of publication. All data generated and analyzed during this study have been deposited in the Dryad database. The raw data can be obtained through the following https://doi.org/10.5061/dryad.ngf1vhj3v.
