# Supplementary material for: Gut Bacteria Improve Depressive Symptoms by Degrading Cortisol into Androgen
Source: Adv Sci (Weinh). 2026 Jan 8;13(11):e08468. doi: 10.1002/advs.202508468 (PMC12931197; doi:10.1002/advs.202508468)
Supplement: Supplementary file 1 — Supporting Information [file ADVS-13-e08468-s002.docx]

Supporting Information

**Gut bacteria improve depressive symptoms by degrading cortisol into androgen**

*Xiong Wang#, Qing Wu#, Hao-Long Zeng#, Yin Shen, Hong-Han Zhang, Jun Gong, Qi Zhang, Jia-Zhao Xie, Da-Wei Ye*, Zi-Yong Sun*, Zhong-Chun Liu*, Li-Ming Chen*, Di Li**

**
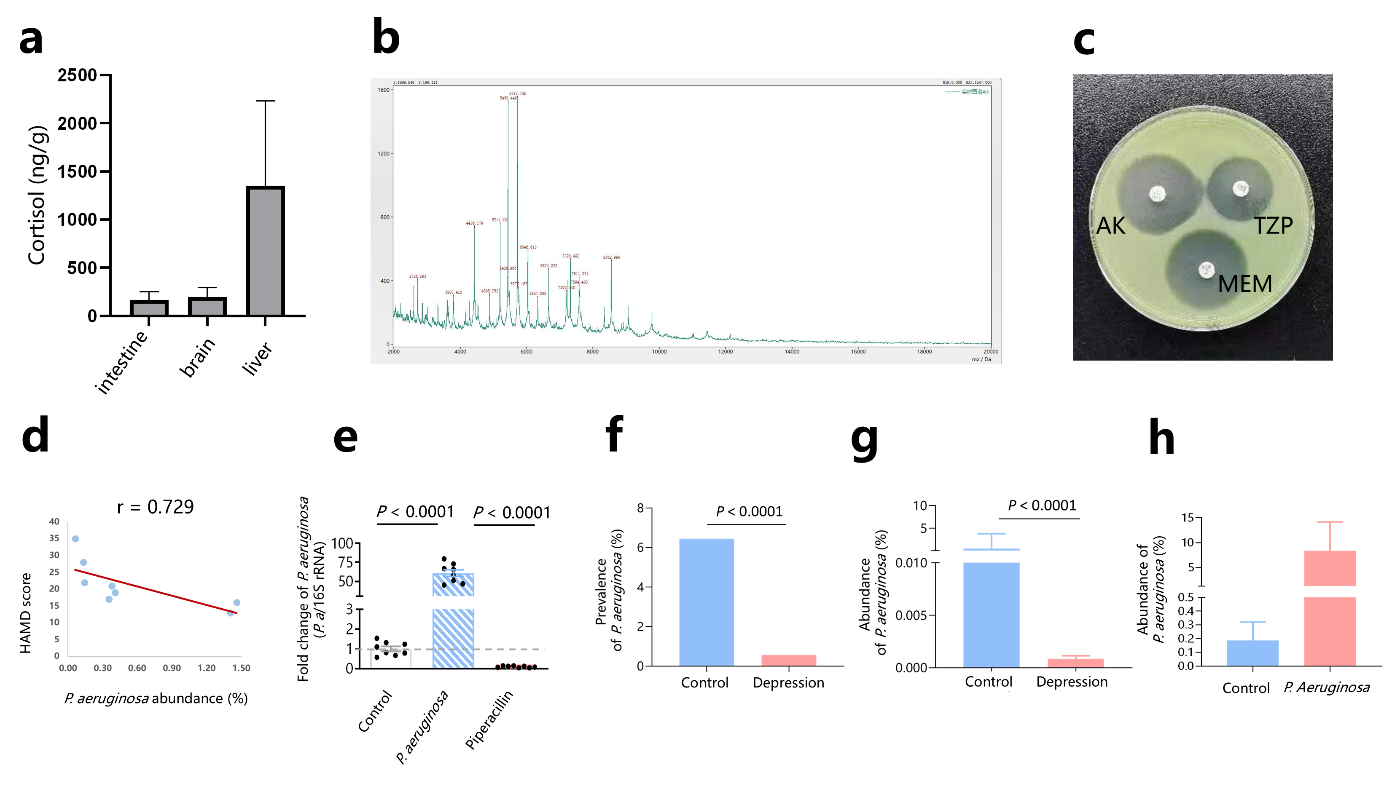
**

**Figure S1**

**Characteristic and prevalence of *P. aeruginosa* Tongji**. **a**, After cortisol treatment, cortisol in gut, brain and liver was 162.77 ± 85.56 ng/g, 197.57 ± 95.87 ng/g and 1349 ± 881.97 ng/g, respectively. **b**, Fingerprint spectrum of *P. aeruginosa* Tongji in MALDI-TOF/TOF. **c**, Drug sensitivity test of *P. aeruginosa* Tongji. AK, MEM and TZP have a lager inhibition zone than their standards for sensitive, indicating that the P. aeruginosa Tongji are sensitive to AK, MEM and TZP. AK: amikacin. MEM: meropenem. TZP: piperacillin. **d**, *P. aeruginosa Tongji* abundance was significantly related to depressive symptom (Hamilton Depression Scale score). r = 0.729, *P* < 0.05. **e**, The colonization and efficacy was measured by *P.aeruginosa*-specific qPCR and normalized to faecal bacteria 16S rRNA gene. **f**, Prevalence of *P. aeruginosa* Tongji in healthy individuals and patients with depression. **g**, Abundance of *P. aeruginosa* in healthy individuals and patients with depression. **h**, Abundance of *P. aeruginosa* in small intestine. Data are representative of at least three independent experiments, n = 10 mice per group. Data are mean ± S.D. One-way ANOVA followed by post hoc Tukey multiple comparison test.


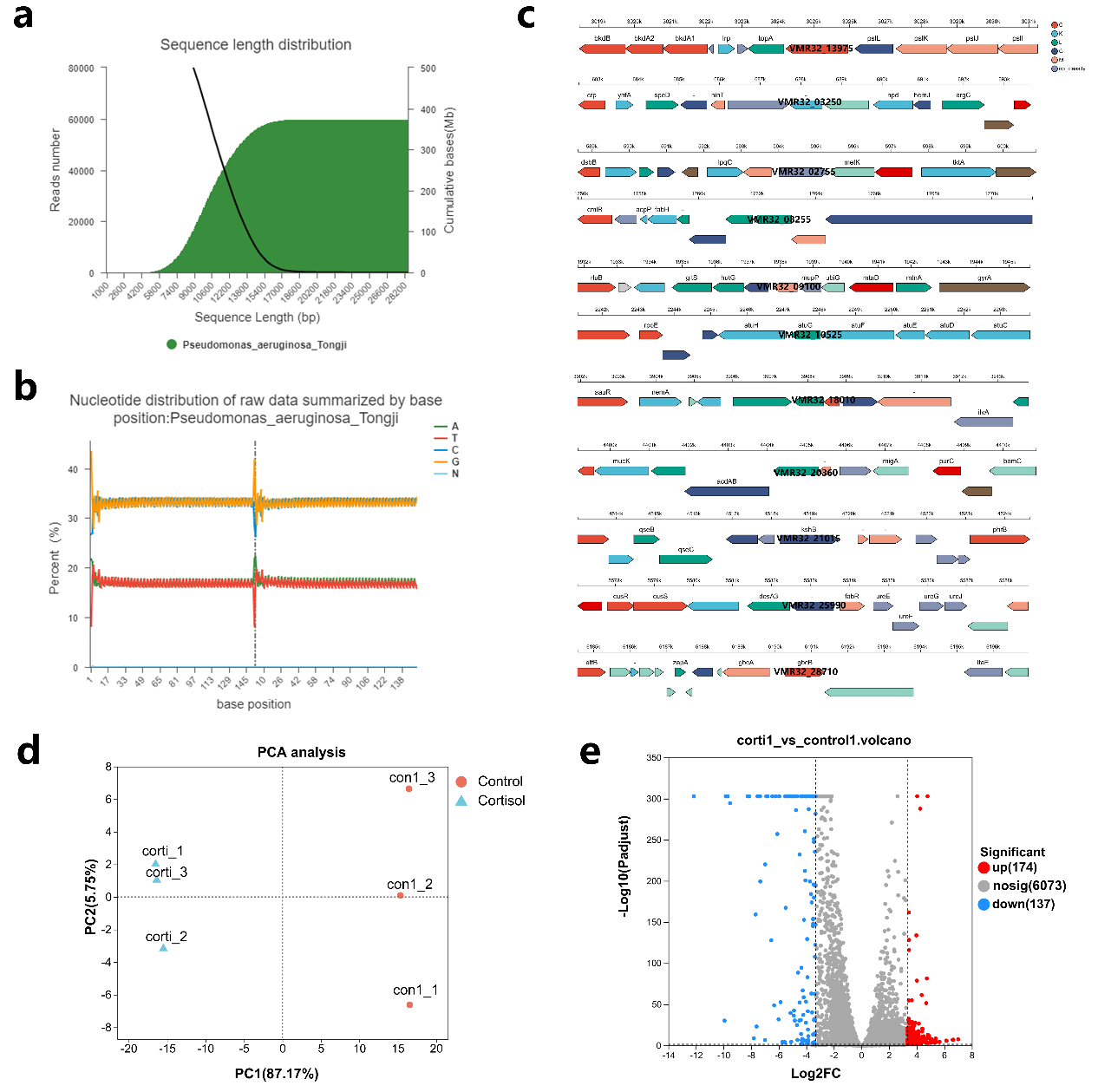


**Figure S2**

**Whole-genome sequencing. a**, Distribution of the length of third-generation reads. Clean Reads length distribution chart. The horizontal axis represents the length of sequenced reads, and the vertical axis represents the number of reads of different lengths. The black curve represents the change in the number of reads as the length of reads increases, whereas the green curve represents the cumulative change in the total base number as the length of reads increases. The green area shows no practical significance. **b**, Raw Reads Base Composition Distribution Map. Distribution map of base composition before quality control. The abscissa is the base coordinate of reads, which represents the sequential arrangement of bases from 5′ to 3′ on reads; the vertical axis represents the percentage of all reads at the sequencing positions A, C, G, T, and N, with different bases represented by different colours. The starting position of the sequence is connected to the primer junction for sequencing, so A, C, G, and T will fluctuate at the starting end and tend to stabilize later. The lower the proportion of the fuzzy base N, the fewer unknown bases and the less affected the sequencing sample is by the system AT preference. **c**, The 11 genes related to steroid metabolism according to annotations in the Swiss-Prot database. The length and direction of the arrows on the map represent the length and coding direction of the genes, respectively; the colour of the arrow represents the COG classification of genes. **d**, PCA analysis of transcriptomics. **e**, Volcano map of the differentially expressed genes in *P. aeruginosa* Tongji with and without 1 g L^-1^ cortisol.


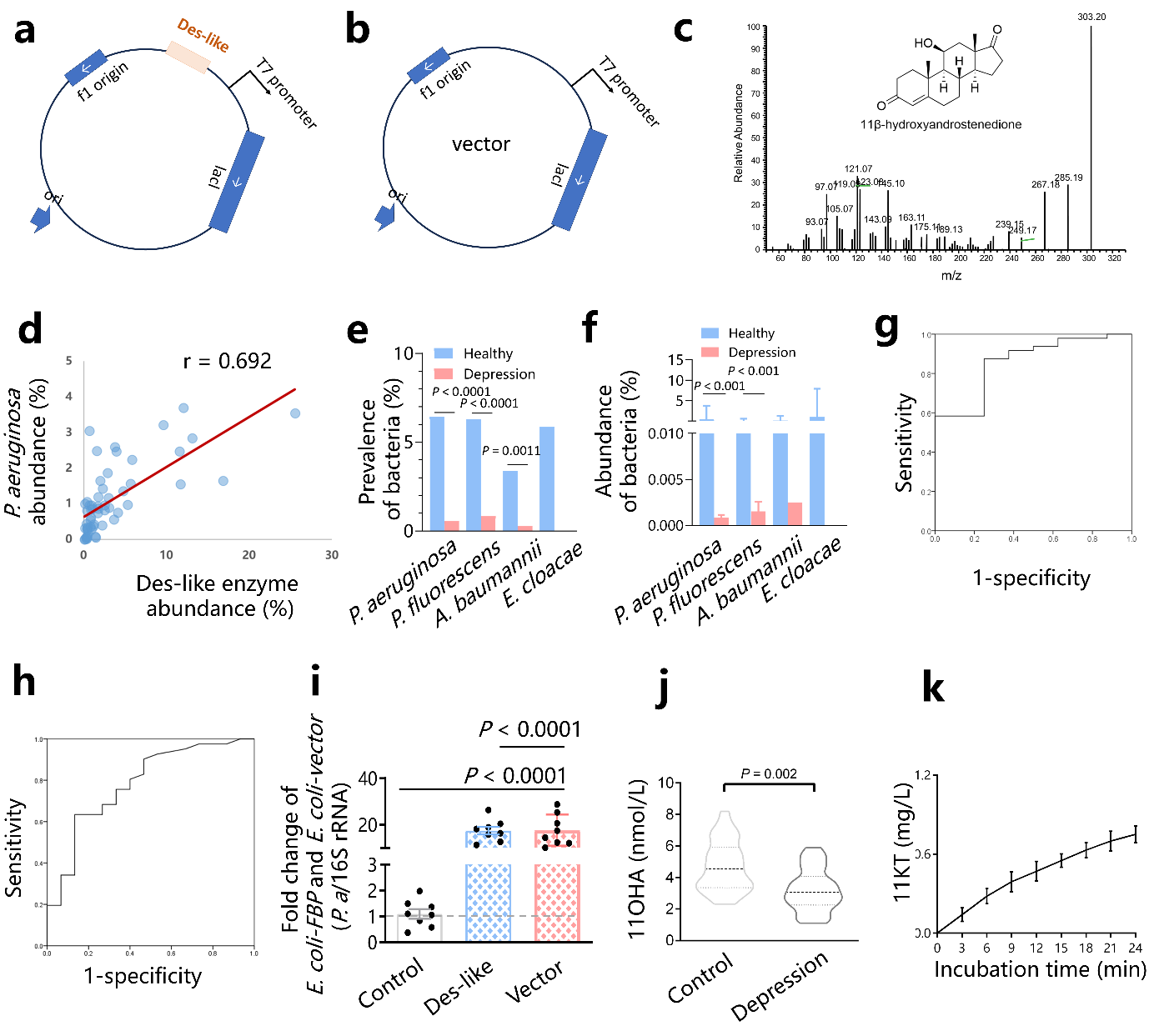


**Figure S3**

**Prevalence of des-like bacteria. a,** des-like enzyme gene inserted into the pET28a plasmid. **b**, Vector pET28a plasmid. **c**, Mass spectrum analysis of 11OHA4. **d**, *P. aeruginosa* abundance was significantly related to des-like enzyme abundance. r = 0.692, *P* < 0.05. **e**, Prevalence rates of *P. aeruginosa*, *P. fluorescens*, *A. baumannii*, and *E. cloacae* in healthy individuals and patients with depression. **f**, Abundances of *P. aeruginosa*, *P. fluorescens*, *A. baumannii*, and *E. cloacae* in healthy individuals and patients with depression. P value of abundances of *A. baumannii*, and *E. cloacae* cannot be calculated due to sample size < 2. **g**, ROC curve was performed to explore the discriminative ability of des-like enzyme abundance in individuals with depression. **h**, independent validation cohort (n = 56) was tested to explore the discriminative ability of des-like enzyme abundance in individuals with depression. **i**. The colonization was measured by *E. coli*-specific qPCR and normalized to faecal bacteria 16S rRNA gene. **j**, 11OHA4 levels in healthy was higher than that in patients with depression. **k**, brain tissue homogenate was incubated with 11OHA4 and the 11KT was detected. Data are representative of at least three independent experiments. Data are mean ± S.D. One-way ANOVA followed by post hoc Tukey multiple comparison test.


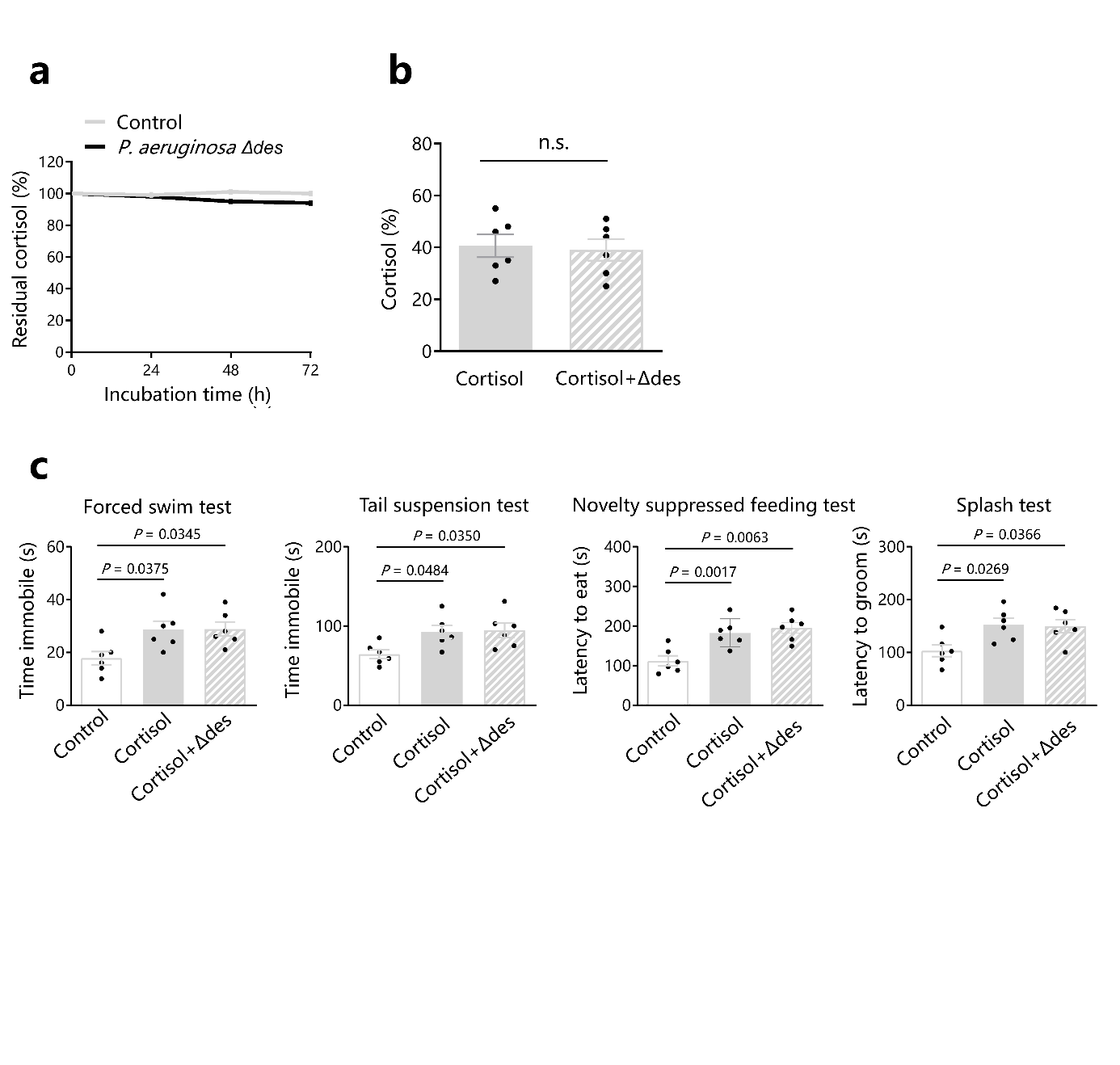


**Figure S4**

**The effect of Δdes-like on corisol and behavior. a,** The des-like gene knockout strain *P. aeruginosa* Δ*des* was incubated with cortisol *in vitro*. No obvious degradation was observed. **b**, Serum cortisol was detected in cortisol treated mice (n = 6) and cortisol treated mice gavaged with *P. aeruginosa* Δ*des* (n = 6). c, Behavior test in cortisol treated mice and cortisol treated mice gavaged with *P. aeruginosa* Δ*des*. Data are mean ± S.D. One-way ANOVA followed by post hoc Tukey multiple comparison test.
